# Obstetric sonographers’ second victim experiences following diagnostic error: implications for wellbeing, support and patient safety

**DOI:** 10.1177/1742271X261489049

**Published:** 2026-09-29

**Authors:** Elaine Gardiner, Julie Roberts, Evelyn McElhinney, Thelma Okey-Adibe, Morgyn Kay

**Affiliations:** 1 Glasgow Caledonian University, UK

**Keywords:** obstetric ultrasound, obstetric sonographer, diagnostic error, second victim phenomenon, sonographer wellbeing, patient safety, support systems, just culture

## Abstract

**Introduction:**

Diagnostic errors in obstetric ultrasound can have devastating consequences for mothers and babies. While patients are the primary victims, healthcare professionals involved may experience psychological harm as ‘second victims’. Obstetric sonographers work in high-risk environments where diagnostic errors can occur; however, their experiences and perceptions of support are underexplored. This study investigated sonographers’ experiences following diagnostic error, focusing on perceived support.

**Methods:**

This qualitative study used a mixed-methods sequential explanatory design within an abductive pragmatic approach. A survey (n = 8) provided context but was limited for quantitative analysis; therefore, qualitative findings are reported. Eight obstetric sonographers with experience of diagnostic error were recruited via purposive and snowball sampling. Semi-structured interviews were conducted remotely, transcribed verbatim and analysed using reflexive thematic analysis. Rigour was enhanced through reflexivity, audit trails and supervisory review.

**Findings:**

Five themes were identified. Participants described experiences consistent with the second victim phenomenon, including emotional distress, loss of confidence, burnout and trauma-like symptoms. While strong professional pride was evident, high workload and emotional demands were emphasised. Unique challenges included delayed awareness of error, caring for both mother and baby and lack of opportunity for apology or closure. Peer support was valued most, while managerial and organisational support was often perceived as inadequate or procedural. Participants called for learning-orientated, just cultures.

**Conclusion:**

Obstetric sonographers experience substantial psychological impact following diagnostic error, shaped by emotional and organisational factors. Greater recognition and trauma-informed, learning-focused support systems are needed to promote wellbeing, patient safety and workforce sustainability.

## Introduction

Healthcare professionals are held to exacting standards of excellence in delivering high-quality care, with no anticipation of error. However, healthcare systems inherently involve some degree of human error.^1^ In antenatal obstetric care, diagnostic errors can have devastating consequences for both the mother and baby, including serious morbidity, long-term disability and even death.^2^ Misdiagnosis and delays are recognised contributors to preventable harm in obstetric practice, including errors in ultrasound interpretation and diagnosis.^3^

UK data highlight the persistent challenge of detecting congenital heart disease (CHD) during the 20-week mid-trimester screening scan. CHD remains one of the most commonly undiagnosed health conditions and is a leading cause of infant morbidity and mortality.^4^ Despite national initiatives to enhance cardiac ultrasound training, antenatal detection rates have plateaued at approximately 50% for the past 7 years, with significant regional variation ranging from 15% to 67%.^5^ Consequently, obstetric sonographers are at risk of making a diagnostic error and missing or misinterpreting a significant finding during their career. Given the significant clinical consequences of missed fetal cardiac abnormalities, sonographers may experience considerable professional and emotional burden following diagnostic error.

Diagnostic errors in ultrasound arise from multiple sources. The World Health Organization (WHO)^6^ defines diagnostic error as a missed, delayed, or incorrect diagnosis. As ultrasound is subjective and highly operator-dependent, it is more prone to errors than other imaging modalities.^7^ Errors may occur when detectable structural abnormalities are overlooked or when measurements and interpretations are inaccurate, potentially leading to misdiagnosis and possible mismanagement of the pregnancy. Systemic factors such as workload, environmental pressures, technical limitations and patient body habitus contribute to these errors, in addition to cognitive factors including perceptual and interpretation biases.^8,9^ These factors highlight that diagnostic error often arises from complex interactions between individual, technical and organisational systems rather than isolated practitioner failings.

While patients and their families are the primary victims of adverse events or errors, there is growing recognition that healthcare professionals involved in errors may experience profound emotional and psychological harm as ‘second victims’. First described by Wu,^10^ following the wider patient safety movement initiated by *To Err is Human* (1999), the second victim phenomenon refers to distress experienced by healthcare professionals following involvement in an unintended error or adverse event, and it has been widely studied in medicine, nursing and other healthcare disciplines. These reactions may include guilt, self-doubt, shame and symptoms consistent with post-traumatic stress.^1^ Obstetric sonographers may be particularly vulnerable to these responses due to the emotional context of pregnancy care, real-time decision-making and their responsibility towards both mother and baby. Despite increasing awareness, research suggests that the needs of second victims are often overlooked and that levels of organisational support vary greatly, influencing the severity and duration of the distress experienced.^11^ However, despite the extensive second victim literature across healthcare professions, there has been little recognition of this issue within ultrasound practice, and the experiences of obstetric sonographers remain largely unexplored. This study seeks to address this long-standing gap in the literature.

Although obstetric sonographers work in a high-risk environment, where evidence demonstrates errors are possible and sometimes unavoidable, there are no studies exploring the experiences of the second victim phenomenon or support in place for sonographers following an adverse outcome. This gap in evidence base highlights the importance and timeliness of this research and provided the rationale for this study. Understanding these experiences is important to inform workforce wellbeing strategies and patient safety initiatives within obstetric ultrasound services.

This study explores the views and experiences of second victim obstetric sonographers following diagnostic error, with particular focus on their perceptions of the support they received. The study was guided by two central research questions designed to examine both the personal impact of error and the support systems available in clinical practice:

**Research Question 1:** What are the views and experiences of second victim obstetric sonographers following making a diagnostic error?
**Research Question 2:** What are second victim obstetric sonographers’ perceptions of support?

## Methods

### Study design

An abductive, pragmatic approach was taken to develop a rich understanding of obstetric sonographers’ experiences as second victims following a diagnostic error. Pragmatism was selected to explore both experiential and practice-orientated aspects of the phenomenon, while abductive reasoning supported iterative interpretation between participant experiences and existing theory.

This study formed part of a wider mixed-methods sequential explanatory design. Although a survey was conducted in an initial phase, the small sample size limited meaningful quantitative analysis; therefore, this paper focuses on the qualitative findings, with survey data used to inform and contextualise the analysis. The qualitative interviews provided the richest insights into participants’ experiences.

### Ethical approval

Ethical approval for this research was granted by an institutional research ethics committee (Code: HLS/PSWAHS/20/220) and obtained from Glasgow Caledonian University’s School of Health and Life Sciences. To ensure confidentiality, participants were assigned pseudonyms during transcription and reporting.

### Sampling strategy

Purposive, convenience and snowball sampling were used to recruit eight obstetric sonographers with experiences of making a diagnostic error. Eligible participants were qualified obstetric sonographers currently or previously practising in the United Kingdom or Ireland who self-identified as having experienced involvement in a diagnostic error. Recruitment was challenging, as sonography is not a protected title and no single national register exists for identifying obstetric sonographers. Given the unsuitability of traditional mailing lists and the restrictions on face-to-face engagement during the COVID-19 pandemic, participants were recruited through study advertisements circulated on Twitter (X) and Facebook, enabling broad reach to sonographers across the United Kingdom and Ireland. All participants were emailed a participant information sheet, interview guide (see Supplementary Material 1) and consent form and informed consent was obtained.

### Data collection

In-depth semi-structured interviews were conducted via Microsoft Teams by a member of the research team. An interview guide (see Supplementary Material) was informed by the work of Scott et al.^12^ and adapted for the obstetric ultrasound context. It explored the diagnostic error event, personal and professional impact, stress responses, support experiences and physical and psychological symptoms. A pilot interview was undertaken to refine both the guide and interview process. Interviews lasted between 28 and 43 min.

### Data analysis

Interviews were audio- and video-recorded and supplemented with field notes to support reflection and analysis. Recordings were transcribed verbatim by the lead researcher, anonymised and checked for accuracy through comparison of the transcripts against the original recordings, with any discrepancies corrected. Reflexive field notes were maintained throughout data collection and analysis to consider how researcher perspectives and professional assumptions may have influenced interpretation. NVivo and Microsoft Excel were used to manage and organise the data set.

Data were analysed using Braun and Clarke’s six-phase thematic analysis.^13^ Both deductive (provisional) and inductive (in vivo and emotion-based) coding approaches were applied, allowing predefined concepts and data-driven insights to inform theme development. Themes were iteratively refined, defined and named. Theme development involved continuous movement between coded extracts, the full dataset and existing literature to identify both semantic and latent patterns of meaning. Regular discussions between the researchers and review of datasets enhanced interpretive rigour. The final analytical process resulted in the identification of themes representing obstetric sonographers’ experiences following diagnostic error.

### Trustworthiness and triangulation

Trustworthiness was strengthened through credibility, dependability, transferability and confirmability measures, including an audit trail, reflexive journalling, supervisory review and integration of multiple data sources.^13^ While a survey was undertaken as part of the wider mixed-methods study, the small sample size limited quantitative analysis. Therefore, in this paper, the survey data were used primarily to inform and contextualise the qualitative findings rather than for formal triangulation. Credibility was enhanced through comparison of interview findings with survey responses, maintenance of an audit trail and regular supervisory discussion throughout analysis.

## Findings

### Participant characteristics

All 8 participants were female obstetric sonographers aged 31–60 years, working in urban settings across England (n = 3), Scotland (n = 2) and Ireland (n = 3). Half had radiography backgrounds (n = 4) and half midwifery (n = 4). All held postgraduate qualifications, and their experience since qualification in obstetric ultrasound ranged from 2 years to greater than 26 years (mean 15 years, estimated from midpoints of banded categories). The number of weekly scan sessions ranged from 0 to 11+, and the number of sessions spent training, supervising or mentoring student sonographers ranged from 0 to 4 per week; participants reporting zero sessions (n = 2) had recently transitioned into education or training-focused roles while maintaining professional registration. Participant characteristics are reported in Table 1.

**Table 1.** Participant Characteristics.

Variable	N (%)
Age	
31–40	2 (25%)
41–50	4 (50%)
51–60	2 (25%)
Gender	
Female	8 (100%)
Professional background	
Midwife	4 (50%)
Radiographer	4 (50%)
Work location	
Urban	8 (100%)
Years of obstetric ultrasound experience	
0–5	1 (12.5%)
6–10	2 (25%)
11–15	1 (12.5%)
16–20	1 (12.5%)
21–25	1 (12.5%)
26+	2 (25%)
Years in previous professional role	
3	1 (12.5%)
4	1 (12.5%)
5	2 (25%)
6	2 (25%)
9	2 (25%)
Level of postgraduate education	
Postgraduate diploma	1 (12%)
Master’s degree	7 (88%)
Scan sessions per week	
0	2 (25%)
3–4	2 (25%)
5–6	1 (12.5%)
7–8	1 (12.5%)
9–10	1 (12.5%)
11+	1 (12.5%)
Training sessions per week	
0	2 (25%)
1–2	4 (50%)
3–4	2 (25%)

Reflexive thematic analysis identified five themes representing obstetric sonographers’ experiences following diagnostic error: Second Victim Experience; Sonographer Professional Pride and Pressures; Challenges Unique to an Obstetric Sonographer; Support for Second Victims; and Organisational Learning, Support and Just Culture. Detailed themes, subthemes and illustrative participant quotations are presented in Supplementary Table 2.

#### Second victim experience

Participants described substantial psychological and professional consequences following diagnostic error. Feelings of guilt, remorse, self-doubt and loss of confidence were evident across accounts and often persisted long after the incident. Participants described both perceptual and documentation errors, several of which resulted in significant maternal or fetal harm. The seriousness of these outcomes frequently intensified feelings of personal responsibility, guilt and grief.

One participant described questioning both her competence and future in the profession:

I very much doubted my skill set . . . I questioned my capabilities . . . I questioned even getting out of the profession. (Emma)

Emotional responses frequently extended beyond the immediate aftermath of the event. Participants reported intrusive thoughts, sadness and ongoing self-doubt.

I felt awful . . . I felt guilty. I felt as if I was a terrible person and I’d done a terrible, unforgivable thing. (Julia)

Several participants also described symptoms consistent with unresolved trauma and burnout, including intrusive memories and flashbacks.

Flashbacks. Yep, I thought about the issue for quite a while afterwards. (Jemma)

The emotional impact frequently extended beyond the immediate aftermath of the event. Several participants continued to reflect on incidents many years later, suggesting unresolved trauma.

Fifteen years later . . . I still think about it. (Alison)

#### Sonographer professional pride and pressures

Participants expressed considerable pride in their profession and viewed obstetric ultrasound as a uniquely demanding area of practice.

I think it’s such a unique job. (Sarah)

Obstetric ultrasound was described as requiring a combination of technical expertise, autonomous decision-making and emotional resilience. Participants frequently described feeling a strong sense of responsibility towards patients and their unborn babies. However, this pride was accompanied by considerable pressure. Participants described high workloads, limited professional recognition and the emotional challenge of moving rapidly between routine and distressing encounters.

The days are so busy that you’re running in, you’re running out, you’re trying to get things sorted . . . (Alison)

The requirement to balance diagnostic responsibilities with sensitive communication was perceived as particularly challenging.

There is no other job that on the hoof you have to explain to that person on the bed what you’ve found that’s gonna rock their world. You’ve got to deal with your own emotions at the same time. (Sarah)

Participants felt these pressures intensified the impact of diagnostic error and contributed to emotional exhaustion.

#### Challenges unique to an obstetric sonographer

Delayed awareness of error emerged as a distinctive challenge. Participants commonly learned of errors months or years after the original event, often through governance investigations, complaints procedures or legal proceedings.

Everybody in the department knew . . . including the office staff . . . before the person that had made the mistake. (Katie)

Several participants expressed frustration that concerns progressed through multiple organisational processes before they themselves were informed. One participant described feeling excluded from processes surrounding the error, explaining that information had passed through multiple organisational layers before reaching her, leaving her feeling ‘like pond life at the bottom of the pool’. This sense of exclusion contributed to feelings of powerlessness and detachment from organisational learning and investigation processes.

Last year . . . I received an e-mail from the hospital lawyers to say that I was expected to respond to a placenta accreta that was missed. (Jill)

Participants also described being expected to continue scanning immediately after learning of an error, often with little opportunity for emotional processing or support.

You’re just devastated, so you don’t have that immediate time to process. And you’re just holding it all in and then feeling teary at the same time . . . and then you wonder if you’re actually missing things because you’re not completely focused, because you keep thinking again to that news. (Amy)

Participants further described the emotional burden associated with caring for both mother and baby. Adverse outcomes frequently generated feelings of grief that extended beyond professional concern.

I was heartbroken for the family . . . I still know the month and the year and I still know the baby’s name. (Emma)

Several participants highlighted the absence of any meaningful opportunity to apologise to affected families or achieve closure.

I would have loved to have apologised to them. (Amy)

Participants felt that delayed feedback, responsibility for two patients and lack of closure collectively intensified second victim experiences.

#### Support for second victims

Experiences of support varied considerably. Peer support was consistently identified as the most valuable source of reassurance because colleagues understood the realities of sonographic practice.

They’d been in similar situations, so they understood how you felt, how the guilt, all the feelings that you had, because they had been there before. (Amy)

I definitely had better peer support . . . they were like ‘you’re a great sonographer’. (Jemma)

Practical peer support was also evident, with colleagues taking over scan lists and encouraging staff to take time away from clinical duties immediately after learning of an error. In contrast, managerial responses were often perceived as procedural rather than supportive.

I got no support from my manager. (Jill)

I feel it is a bit of a tick box exercise for them . . . ‘I’ve informed them,’ tick the box. (Emma)

Participants also reported a lack of clear organisational pathways for supporting staff following diagnostic error.

At the NHS, I don’t think we have very clear pathways for discussing this kind of event. (Amy)

Many participants described feeling isolated during investigations and believed organisational responses often prioritised process over emotional wellbeing.

#### Organisational learning, support and just culture

Participants consistently advocated for more open discussion of error and greater emphasis on learning rather than blame. Organisational cultures were frequently characterised as blame-orientated rather than learning-focused, which participants perceived as creating barriers to emotional recovery, openness and shared learning.

As Julia explained:

Creating an open culture . . . an open community of practice and learning . . . you will make mistakes . . . we are all human.

Participants believed that greater transparency and discussion of mistakes could reduce shame, support professional development and prevent repeated errors.

The same mistakes were being made over and over . . . no one learns . . . it’s because of the shame. (Julia)

Structured support systems, protected time for reflection and opportunities for debriefing were viewed as important priorities following adverse events.

We need a bit of time out . . . an opportunity to discuss what to do next. (Amy)

Participants also emphasised the importance of training in emotional resilience, communication and learning from errors.

Training was very much practical training . . . not emotional intelligence . . . a bereavement course helped me with learning to cope with things. (Emma)

Greater involvement of sonographers in audit, investigation and learning processes was viewed as important for improving both workforce wellbeing and patient safety.

## Discussion

This study provides the first qualitative insight into obstetric sonographers’ experiences as second victims following diagnostic error.

## Key findings

The findings demonstrate that diagnostic errors are associated with profound and sometimes long-lasting psychological, emotional and professional consequences. Support following diagnostic error was perceived as largely informal, with peer support valued most and managerial and organisational support frequently described as inconsistent, procedural, or absent. These findings raise important implications for workforce wellbeing, patient safety, and organisational culture in obstetric ultrasound practice.

### Second victim impact in obstetric sonographers

This study confirms that obstetric sonographers experience significant second victim impact, manifested predominantly through psychological and emotional distress. These experiences mirror findings across second victim research in nursing and medicine, where emotional and psychological symptoms consistently outweigh physical responses.^14–16^

The enduring nature of participants’ accounts suggests that second victim experiences may persist long-term, particularly when emotional processing, peer support and organisational acknowledgement are lacking.^17^ Intrusive thoughts, hypervigilance, sleep disturbance and persistent anxiety described by participants are consistent with symptoms associated with burnout and trauma responses identified in second victim literature.^18,19^

Several factors specific to obstetric ultrasound practice may contribute to the prolonged nature of these trauma responses. Obstetric sonographers often work in relative professional isolation, undertaking autonomous real-time diagnostic decision-making with limited immediate peer review. Unlike many acute healthcare incidents, diagnostic errors in ultrasound may remain undisclosed for months or years, delaying opportunities for emotional processing, learning and closure. In addition, repeated exposure to pregnancy care and fetal outcomes may foster strong emotional attachment to patients and pregnancies, intensifying feelings of guilt and grief when adverse outcomes occur.^3^ The often hidden and retrospective nature of diagnostic error may further compound rumination, self-blame and fear of recurrence.^1,9,12^

While this study did not formally diagnose post-traumatic stress disorder, several participants reported symptom clusters overlapping with recognised trauma responses. These findings support concerns that when second victim experiences remain unresolved, there are heightened risks of long-term psychological harm and impaired professional functioning.^16,17^

### Professional impact and workforce implications

The professional consequences of diagnostic error were profound. Participants described reduced confidence, hypervigilant scanning practice, avoidance of complex cases, difficulty concentrating and strong intention to leave the profession. These findings align with second victim studies demonstrating associations between adverse events, cognitive disruption and turnover intentions.^16,20,21^ These findings are particularly concerning within the context of ongoing sonographer workforce shortages and increasing demand within maternity services.

Despite substantial emotional distress, absenteeism was low. This contrasts with some second victim literature reporting increased absenteeism, turnover intentions and withdrawal from practice following adverse events,^11,16,20^ but mirrors findings by Brunelli et al.^22^ and may reflect a strong sense of duty, professional identity or guilt. However, continuing to work while emotionally distressed raises concerns regarding presenteeism and patient safety, as cognitive load, fatigue and anxiety may increase error risk.^23^

While some sonographers reported increased vigilance and reflection on practice, these experiences were not universal. This variability supports evidence that positive adaptation following adverse events is contingent upon timely and appropriate support, rather than occurring inherently.^24^ Without adequate support structures, the risk of burnout, disengagement and workforce attrition is amplified.

### The unique context of obstetric ultrasound practice

This study highlights features of obstetric ultrasound practice that distinctly shape sonographers’ second victim experiences. Unlike many healthcare errors identified immediately, ultrasound diagnostic errors are often detected retrospectively; at a subsequent scan, delivery or even years later, limiting opportunities for immediate learning and emotional resolution.^25^ This delay may exacerbate rumination, uncertainty and fear of recurrence.

In addition, obstetric sonographers frequently communicate diagnostic findings in real time, often without structured training in delivering distressing news. This places considerable emotional and cognitive demands on practitioners and contributes to burnout risk.^26,27^ The requirement to rapidly transition between routine reassurance and delivery of distressing news reflects significant emotional labour within obstetric ultrasound practice. Errors may affect both mother and baby, intensifying moral distress, guilt and grief, a pattern similarly observed among obstetricians and midwives involved in traumatic births.^28^

A further challenge unique to sonographers is the limited opportunity to apologise or achieve closure with patients following an error. Despite professional duty of candour requirements,^29,30^ sonographers rarely have direct follow-up contact with affected families. Literature suggests that apologies and face-to-face dialogue can support healing for both patients and clinicians^31,32^ and the absence of such opportunities may prolong emotional distress and unresolved guilt among sonographers.

### Support gaps and organisational culture

The prominence of peer support in this study is consistent with extensive second victim literature.^19,33^ Support from colleagues with shared professional understanding helped normalise distress and reduce isolation. However, reliance on informal peer support highlights a critical lack of structured organisational response.

Participants’ accounts of inconsistent managerial support echo findings that managers may focus on investigation processes while overlooking emotional impact.^34^ Institutional support was frequently rated as poor, consistent with evidence that healthcare organisations often fail to recognise or respond effectively to second victim needs.^21^

Participants’ experiences of blame, shame and fear of judgement contrast with the principles of a *Just Culture*, which emphasises learning, fairness and system accountability.^35,36^ Failure to implement just culture principles risks reinforcing silence, fear and repeated error, while undermining staff wellbeing and safety culture.

## Implications for ultrasound practice

While detailed intervention development is beyond the scope of this paper, the findings clearly support the need for trauma-informed, system-level support strategies embedded within obstetric ultrasound services. Evidence from both this study and wider second victim literature suggests that support should operate across several key areas.

Education and training should include greater awareness of diagnostic error, second victim phenomena, emotional resilience and communication skills within sonography education and continuing professional development. Several participants highlighted the need for additional preparation in emotional intelligence, bereavement communication and delivery of difficult news.

Peer support systems were consistently identified as the most valued sources of support following diagnostic error. Development of structured peer support or mentorship pathways may help normalise experiences, reduce professional isolation and facilitate shared learning.

Debriefing opportunities and protected recovery time may also be beneficial. Participants frequently described being expected to continue scanning immediately following disclosure of an error, often without the opportunity for emotional processing or reflective discussion. Formal debriefing systems may support both wellbeing and organisational learning.

Leadership and manager training is essential to promote psychologically informed responses to adverse events. Participants frequently perceived managerial responses as procedural rather than compassionate, highlighting the importance of leadership approaches grounded in psychological safety and Just Culture principles.

Finally, access to counselling and psychological support services, including employee assistance programmes and trauma-informed wellbeing support, should be readily accessible for sonographers experiencing prolonged distress following diagnostic error.^12,37,38^

These approaches align with professional guidance advocating learning-orientated cultures, interprofessional engagement and wellbeing-focused leadership.^39,40^ Without structural change, reliance on individual resilience is likely to perpetuate burnout and workforce attrition, particularly within an already constrained sonography workforce.

## Limitations

Several limitations are acknowledged. The sample was small (n = 8) and drawn entirely from female sonographers working in urban settings across the United Kingdom and Ireland, which, together with recruitment via social media, may have introduced self-selection bias and limits transferability of findings to male sonographers, rural services and other healthcare systems. As participation was voluntary, it is possible that sonographers with particularly strong emotional experiences were more likely to come forward and share their experiences. As participation also required self-identification as having made a diagnostic error, those most profoundly affected or conversely those least willing to revisit the experience may be under-represented.

Accounts were retrospective and self-reported and may therefore be subject to recall bias; however, the vividness and consistency of recall, sometimes of events many years earlier, arguably reflect the enduring impact of these experiences rather than diminishing their validity.

Coding and analysis were led by a single researcher, which may introduce interpretative bias, although this was mitigated through reflexivity and regular supervision review. Formal member checking was not undertaken. Consistent with a reflexive thematic analysis approach, emphasis was placed on reflexivity, supervisory discussion and maintenance of an audit trail rather than participant validation of themes. Nevertheless, participant feedback may have provided additional perspectives and strengthened confirmability.

Finally, as the small sample precluded meaningful quantitative analysis, the findings are interpretative rather than generalisable. Future research with larger more diverse samples, including longitudinal designs, would help establish the prevalence, trajectory and modifiable determinants of second victim experiences among sonographers.

## Conclusion

Obstetric sonographers experience substantial second victim distress following diagnostic error, shaped by emotional demands, delayed error recognition and structural constraints of ultrasound practice. While peer support offers important mitigation, inconsistent managerial and organisational responses can compound harm. Evidence from intervention-focused studies highlights the value of structured, compassionate and trauma-informed support strategies, including peer support programmes, facilitated debriefing and organisational learning approaches. These findings highlight the need for greater recognition of second victim experiences within obstetric ultrasound and for organisational approaches that support both workforce wellbeing and patient safety. Embedding such approaches within obstetric ultrasound services may help sustain workforce wellbeing, support retention and promote safer maternity care.

## Supplemental Material

sj-docx-1-ult-10.1177_1742271X261489049 – Supplemental material for Obstetric sonographers’ second victim experiences following diagnostic error: implications for wellbeing, support and patient safety Supplemental material, sj-docx-1-ult-10.1177_1742271X261489049 for Obstetric sonographers’ second victim experiences following diagnostic error: implications for wellbeing, support and patient safety by Elaine Gardiner, Julie Roberts, Evelyn McElhinney, Thelma Okey-Adibe and Morgyn Kay in Ultrasound

sj-docx-2-ult-10.1177_1742271X261489049 – Supplemental material for Obstetric sonographers’ second victim experiences following diagnostic error: implications for wellbeing, support and patient safety Supplemental material, sj-docx-2-ult-10.1177_1742271X261489049 for Obstetric sonographers’ second victim experiences following diagnostic error: implications for wellbeing, support and patient safety by Elaine Gardiner, Julie Roberts, Evelyn McElhinney, Thelma Okey-Adibe and Morgyn Kay in Ultrasound

## References

[1] Nydoo P Pillay BJ Naicker T , et al. The second victim phenomenon in health care: a literature review. Scand J Public Health 2019; 48(6): 629–637. 10.1177/1403494819855506 31405351

[2] NHS Resolution. Annual report and accounts 2024/25 (HC 1065). NHS Resolution, 2025.

[3] Krenitsky NM Perez Urbano I Goffman D. Diagnostic errors in obstetric morbidity and mortality. J Clin Med 2024; 13(14): 4245. 10.3390/jcm13144245 PMC11278303 39064285

[4] NHS England Digital. Diagnostic imaging dataset statistical release. NHS England Digital, 2024.

[5] NICOR. National Cardiac Audit Programme (NCAP): annual report. NICOR, 2025.

[6] World Health Organization. Diagnostic errors: technical series on safer primary care. WHO, 2016.

[7] Di Serafino M Iacobellis F Schillirò ML , et al. Common and uncommon errors in emergency ultrasound. Diagnostics 2022; 12(3): 631. 10.3390/diagnostics12030631 PMC8947314 35328184

[8] Graber M. Diagnostic errors in medicine: a case of neglect. Jt Comm J Qual Patient Saf 2005; 131(2): 106–113. 10.1016/s1553-7250(05)31015-4 15791770

[9] Itri JN Patel SH. Heuristics and cognitive error in medical imaging. AJR Am J Roentgenol 2018; 210(5): 1097–1105. 10.2214/AJR.17.18907 29528716

[10] Wu AW. Medical error: the second victim. BMJ 2000; 320: 726–727. 10.1136/bmj.320.7237.726 PMC1117748 10720336

[11] Burlison JD Quillivan RMS Scott SD , et al. The effects of the second victim phenomenon and work-related outcomes: connecting self-reported caregiver distress to turnover intentions and absenteeism. J Patient Saf 2018; 17(3): 195–199. 10.1097/PTS.0000000000000301 PMC5413437 27811593

[12] Scott SD Hirschinger LE Cox KR , et al. The natural history of recovery for the healthcare provider ‘second victim’. Qual Saf Health Care 2009; 18(5): 325–330. 10.1136/qshc.2009.032870 19812092

[13] Braun V Clarke V. Thematic analysis: a practical guide. Sage, 2022.

[14] Wu AW Shapiro J Harrison R , et al. The impact of adverse events on clinicians. J Patient Saf 2020; 16(1): 65–72. 10.1097/PTS.0000000000000256 29112025

[bibr15-1742271X261489049] Cabilan CJ Kynoch K. Experiences of second victims: a systematic review. JBI Database System Rev Implement Rep 2017; 15(9): 2333–2364. 10.11124/JBISRIR-2016-003254 28902699

[16] Burlison JD Quillivan RR Scott SD , et al. Second victim phenomenon and work outcomes. J Patient Saf 2021; 17(3): 195–199. 10.1097/PTS.0000000000000301 PMC5413437 27811593

[17] Kappes M Romero García M Delgado Hito P. Coping strategies in healthcare providers as second victims. Int Nurs Rev 2021; 68(4): 471–481. 10.1111/inr.12694 34118061

[18] Sachs CJ Wheaton N. Second victim syndrome. StatPearls Publishing, 2025. 34283460

[19] Simms Ellis R Harrison R Sattar R , et al. Support for second victims in healthcare: a systematic review. BMJ Open 2025; 15(2): e087512. 10.1136/bmjopen-2024-087512 PMC12185930 39929502

[20] Mira JJ Carrillo I Lorenzo S , et al. The aftermath of adverse events. BMC Health Serv Res 2015; 15: 151. 10.1186/s12913-015-0790-7 PMC4394595 25886369

[21] Mahat S Lehmusto H Rafferty AM , et al. Impact of second victim distress. J Adv Nurs 2024; 81(9): 5376–5388. 10.1111/jan.16291 PMC12371787 38896051

[22] Brunelli MV Estrada S Celano C. Cross-cultural adaptation of SVEST. J Patient Saf 2021; 17(8): e1401–e1405. 10.1097/PTS.0000000000000497 29733300

[23] Widera E Chang A Chen HL. Presenteeism: a public health hazard. J Gen Intern Med 2010; 25(11): 1244–1247. 10.1007/s11606-010-1422-x PMC2947637 20549378

[24] Zhang C Yang Z Liang Y , et al. Forced growth during patient safety incidents. BMC Nurs 2024; 23: 592. 10.1186/s12912-024-02255-7 PMC11346293 39183276

[25] Rodziewicz TL Houseman B Vaqar S , et al. Medical error reduction and prevention. StatPearls Publishing, 2024. 29763131

[26] Johnson J Arezina J McGuinness A , et al. Breaking bad news in obstetric ultrasound. Ultrasound 2019; 27(1): 55–63. 10.1177/1742271X18816535 PMC6362539 30774699

[27] Hardicre NK Arezina J McGuinness A , et al. Sonographer experiences of difficult news delivery. Radiography 2021; 27(2): 369–376. 10.1016/j.radi.2020.09.015 33077330

[28] Schroeder K Jørgensen JS Lamont RF , et al. Blame and guilt after traumatic childbirth. Acta Obstet Gynecol Scand 2016; 95(7): 735–745. 10.1111/aogs.12897 27072600

[29] Nursing Midwifery Council. Openness and honesty when things go wrong. NMC, 2018.

[30] Health Care Professions Council. The duty of candour. HCPC, 2024.

[31] Robbennolt JK. Apologies and medical error. Clin Orthop Relat Res 2009; 467(2): 376–382. 10.1007/s11999-008-0580-1 PMC2628492 18972177

[32] Aubin DL Soprovich A Carvallo FD , et al. Support after medical error. BMJ Open Qual 2022; 11(4): e002004. 10.1136/bmjoq-2022-002004 PMC9730392 36588324

[33] Finney RE Torbenson VE Riggan KA , et al. Second victim experiences in obstetrics. J Nurs Manag 2020; 29(4): 642–652. 10.1111/jonm.13198 PMC8079544 33113207

[34] Christoffersen JT Rønningstad C. Supporting midwives after adverse incidents. Midwifery 2020; 85: 102669. 10.1016/j.midw.2020.102669 32120330

[35] Reason J. Managing the risks of organizational accidents. Ashgate, 1997.

[36] Boysen PG. Just culture. Ochsner J 2013; 13(3): 400–406. PMC3776518 24052772

[37] Seys D Panella M Russotto S , et al. Second victim support: a narrative review. BMC Health Serv Res 2023; 23: 816. 10.1186/s12913-023-09637-8 PMC10391912 37525127

[38] European Researchers’ Network Working on Second Victims. Second victims support interventions, 2024.

[39] British Medical Ultrasound Society. NHS sonographers: scope of practice survey. BMUS, 2021.

[40] Society and College of Radiographers. Guidance for obstetric ultrasound services. SCoR, 2023.

