# Supplementary material for: Obstetric sonographers’ second victim experiences following diagnostic error: implications for wellbeing, support and patient safety
Source: Ultrasound. 2026 Sep 29:1742271X261489049. Online ahead of print. doi: 10.1177/1742271X261489049 (PMC13627462; doi:10.1177/1742271X261489049)
Supplement: Supplementary file 1 [file sj-docx-1-ult-10.1177_1742271X261489049.docx]

# Supplemental Material: Interview Guide

**The impact on second victim obstetric sonographers following a diagnostic error and support mechanisms: an exploration.**

**Interview Guide**

Start with reminding the participant of this part of the study to ensure that consent is ongoing. Re-affirm that they are able to omit questions, take time to compose themselves or withdraw from the study if they become upset.

**The Event Details**

- Think about an event where you have made a diagnostic error in your ultrasound practice. Please share what you remember as specifically as possible from the moment that it was discovered that something was wrong.
- How did you find out that something was wrong?
- How did you respond to this event?
- How did it impact you?

Interviewer: If not forthcoming by this participant’s recollection, and as appropriate, determine:

- How long ago did this event occur
- Type of event (diagnostic error versus unexpected medical outcome)
- Describe your specific role in the event
- The patient / fetal outcome (no harm, temporary harm, permanent harm, death)

**Professional and personal impact from this experience**

- Thank you for sharing the event details with me. Now I would like to focus on what needs you experienced immediately after this event both personally and professionally.
- What needs were addressed?
- How were these needs addressed?
- What needs did you have that were not addressed?
- What would you recommend for having these needs addressed?

**Participant experiences with, or normal reactions to, stress**

- When you are concerned or stressed about something happening at work, how do you typically manage those types of situations?
- Who do you typically turn to when you need advice or reassurance or support about a work-related issue?
- In your professional training, did you learn how to respond to adverse patient events on a professional and/or personal basis?
- Where do you believe is the best place or approach for departments and staff to learn about how to handle adverse events?

Support structures

- Based on your experience, what would you do differently if you were supporting a peer or colleague going through the same thing you went through?
- How would you describe the environment your place of work in terms of being supportive/helpful versus non-supportive/not helpful after an event that has an emotional impact on a team member?
- What is your advice to us as we plan design for a ‘‘perfect world’’ where the best support/guidance possible is provided when a team member(s) is emotionally impacted following an unexpected outcome or adverse event?
- What else would you like to share about your experience?
- Please review this list of symptoms (below) that some staff have reported. If you experienced any of these, please tell me how that symptom impacted you both personally and professionally.
- Are there any symptoms not on this list that you think should be included on this list?
- Thank you for your time today. Before we finish is there anything more you would like to say about what we have discussed?

**List of Symptoms**

**Physical symptoms**	**Psychosocial symptoms**

Extreme fatigue	Frustration

Sleep disturbances	Decreased job satisfaction

Rapid heart rate	Anger

Increased blood pressure	Extreme sadness

Muscle tension	Difficulty concentrating

Rapid breathing	Flashbacks

Loss of confidence

Grief

Remorse

Depression

Repetitive/intrusive memories

Self-doubt

Return to work anxiety

Second guessing career

Fear of reputation damage

Excessive excitability

Avoidance of patient care area
