# Supplementary material for: Obstetric sonographers’ second victim experiences following diagnostic error: implications for wellbeing, support and patient safety
Source: Ultrasound. 2026 Sep 29:1742271X261489049. Online ahead of print. doi: 10.1177/1742271X261489049 (PMC13627462; doi:10.1177/1742271X261489049)
Supplement: Supplementary file 2 [file sj-docx-2-ult-10.1177_1742271X261489049.docx]

Table 2. Themes and sub-themes with selected participant quotes.

| Themes | Sub-Theme | Participant Quotes |
| --- | --- | --- |
| Second Victim Experience | - Professional impact - Personal impact   - Physical responses   - Emotional responses - Unresolved Trauma and Burnout | *Professional impact*  **Emma:** “I very much doubted my skill set . . . I questioned my capabilities. . . I questioned even getting out of the profession”  **Jill:** “Return to work anxiety . . . and second guessing my career . . . absolutely I want to leave, that's for sure”  **Julia:** “when I went back. . . I’d lost faith in my abilities to a fairly sizable degree.”  **Emma**: Here we call it the brown envelope. . . the letter with the date or time that you're going to appear in court”  *Physical responses*  **Julia:** “I started getting chest pains and pins and needles . . . I had to stop working.”  **Emma:** “fatigue . . . I was exhausted. . . I was shattered”  **Jill:** “at the time I had extreme fatigue, sleep disturbances. . . palpitations . . . muscle tension”  **Sarah:** “rapid heart, right along with going to be sick . . . when you first find out”  *Emotional responses*  **Julia:** “I felt awful . . . I felt guilty. I felt as if I was a terrible person and I'd done a terrible, unforgivable thing.”  **Amy:** “I felt extreme sadness . . . I was very teary”  **Katie:** “extreme sadness, difficulty concentrating, loss of confidence, remorse and those repetitive intrusive memories. . .just thinking about it quite a lot and self-doubt”  *Unresolved Trauma and Burnout*  **Emma:** “This was probably two, three years ago . . . It feels like it was only yesterday. It still feels very… raw”  **Alison:** “Fifteen years later . . . I still think about it”  **Jemma:** “Flashbacks. Yep, I thought about the issue for quite a while afterwards”  **Alison:** “I think that everybody is just exhausted, they're tired. They're burnt out. We did the pandemic . . . everybody is tired” |
| Sonographer Professional Pride and Pressures | - Professional Identity, Pride and Pressure - Professional Recognition - Workload pressures - Communication under pressure | *Professional Identity, Pride and Pressures*  **Alison**: “I personally feel that we're special people within a very specialized area and it does keep everybody very humble.”  **Emma**: “We all get into this job because of our emotions.”  *Professional Recognition*  **Julia**: “There's a lack of understanding or appreciation of what we do”  **Sarah**: “it's sort of a lack of professional recognition in your judgments, in your decision making”  *Workload pressures*  **Alison:** “The days are so busy that you're running in, you're running out, you're trying to get things sorted and it's only when you actually sit down and go, “oh, that was some day!””  **Emma:** “It just gets down to unfortunately the busyness of the departments . . . you're just so busy.”  *Communication under pressure*  **Sarah**: “I think it's such a unique job. . . there is no other no other job that on the hoof . . . you have to explain to that person on the bed what you’ve found that's gonna’ rock their world. You’ve got to deal with your own emotions at the same time”  **Alison**: “When they come in to us, they don't have that warning shot... We get the anger, we get their denial, we get their upset... then you have to put them into a quiet room, get them a cup of tea and go into the next couple and be all smiles. It's like Jekyll and Hyde, you're two personalities...” |
| Challenges Unique to an Obstetric Sonographer | - Delayed Awareness - Expected to carry on - Inability to apologise / lack of closure - Caring for two - Type of error and patient impact | *Delayed Awareness*  **Katie:** “Everybody in the department knew . . . including the office staff . . . before the person that had made the mistake”  **Julia:** “most people working in this job don't have any understanding that for that piece of information to reach me, it must have gone through multiple layers in the hospital, through legal department, through clinical governance and Lead for Obs and Gyn . . . and I had no consciousness of it. It was all happening above my head . . . and it reinforced for me the idea that I was pond life at the bottom of the pool.”  **Jill:** “last year, in the middle of the year, I received an e-mail from the hospital lawyers to say that I was expected to respond to a placenta accreta that was missed.”  *Expected to carry on*  ***Emma****: “ I was sent back in after I was told and I was exhausted . . . I was shattered”*  ***Sarah:*** *“Get back on your horse and get back in your room”*  *Inability to apologise*  **Amy:** “I would have loved to have apologised to them”  *Caring for two*  **Emma:** “I was heartbroken for the family. . . I still know the month and the year and I still know the baby's name”  **Emma:** “I felt I was grieving. . . the six stages of grief . . . and it wasn't even my own baby”  *Type of error and patient impact*  **Jemma**: “It was a low lying placenta which meant she couldn’t have a normal delivery . . . in my documentation I put [the hyphen] the wrong way . . . she went on for induction, and she ended up having a bleed”  **Julia:** “I marked what was actually a low-lying placenta as high anterior. . she ended up with a crash caesarean and hysterectomy.”  **Emma**: “I should have seen it . . hydrops was identified . . . a couple of weeks later fetal demise.”  **Katie**: “I missed an ectopic. . . . just inexperience . . . told her that it was an ongoing pregnancy . . . she went home and ruptured that night.” |
| Support for Second Victims | - Colleague support - Manager support - Institutional support - Friends and family - Supporting other colleagues | *Colleague Support*  **Jemma:** “I definitely had better peer support . . . they were like “you're a great sonographer”  **Amy:** “they'd been in similar situations, so they understood how you felt, how the guilt, all the feelings that you had, because they’d been there before”  **Sarah:** “I have got excellent support . . . because they all know me”  *Manager support*  **Jill:** “I got no support from my manager”  **Jemma:** “they didn't even seem to want to know from my side what had happened.”  **Emma:** “I feel it is a bit of a tick box exercise for them . . . “I've informed them tick the box”  *Institutional support*  **Amy**: “At the NHS, I don't think we have very clear pathways for discussing this kind of event”  **Emma:** “I actually did the EAP [employee assistance programme] . . . because I felt my confidence was lacking and I was suffering from anxiety at nighttime . . . they taught me coping skills”  *Friends and Family*  **Amy:** “you talk about it with your friends and your family, but not in a professional manner. . . they may have that ‘gasp’ reaction of “you could have missed that, that's terrible” . . . and not really understand your job  *Supporting other colleagues*  **Emma:** “ I went and took their patient. . . got them a coffee . . . told them to sit in the room . . . they knew I was here just to get their thoughts together.”  **Jill:** “I feel very ashamed that I can't share . . . younger sonographers aren't able to learn from it”  **Julia:** “the same mistakes were being made over and over. . .no one learns . . . it's because of the shame” |
| Organisational Learning, Support and Just Culture | - Just and Open Culture - Structured Support Systems - Learning and Training | *Just and Open Culture*  **Julia:** “don’t wear a name badge, because if they don’t know who you are they can’t report you”  **Julia**: “creating an open culture, an open community of practice and learning . . . open the dialog, “you will make mistakes, . . .we are all human”  **Jemma:** “I think people need to know that they weren't to blame. . . it happens to everybody. . . you're not on your own”  **Alison:** “sharing of our mistakes and learning from them rather than being hidden away”  *Structured Support Systems*  **Amy:** “time to talk about it and to discuss feelings in a more professional way . . . rather than just between patients crying to your colleague. . . we need a bit of time out. . . an opportunity to discuss what to do next”  **Julia:** “make sure that you learn from serious incidents. . . or it's a missed opportunity for professional development . . . for safety.”  **Jill: “**guidance on what can we do now that this has happened? What is the outcome and how can we improve it”  *Learning and Training*  **Julia:** “I want the sonographers to be part of audit and MDT investigations, to actually have a voice and to have a stake in the department. That way . . .that you don't have this erroneous idea that no one else makes mistakes”  **Alison:** “It's reassuring to hear their stories that they've done it . . . because when it happens, you think it's just you”  **Emma:** “training was very much practical training. . . not emotional intelligence. . . a bereavement course helped me with learning to cope with things.”  **Jemma:** “for some people, pregnancy isn't straightforward and delivering bad news is something I would like to do more on” |
